# Grouping, Spectrum–Effect Relationship and Antioxidant Compounds of Chinese Propolis from Different Regions Using Multivariate Analyses and Off-Line Anti-DPPH Assay

**DOI:** 10.3390/molecules25143243

**Published:** 2020-07-16

**Authors:** Xiasen Jiang, Linchen Tao, Chunguang Li, Mengmeng You, George Q. Li, Cuiping Zhang, Fuliang Hu

**Affiliations:** 1 College of Animal Science, Zhejiang University, Hangzhou 310058, China; jxsen@zju.edu.cn (X.J.); 11817025@zju.edu.cn (L.T.); mmyou@zju.edu.cn (M.Y.); lgzcplyx@aliyun.com (C.Z.); 2 NICM Health Research Institute, Western Sydney University, Westmead, NSW 2145, Australia; c.li@westernsydney.edu.au (C.L.); g.li@westernsydney.edu.au (G.Q.L.)

**Keywords:** propolis, antioxidant activity, spectrum–effect relationships, cluster analysis, principal component analysis, multiple linear regression analysis

## Abstract

49 samples of propolis from different regions in China were collected and analyzed for their chemical compositions, contents of total flavonoids (TFC), total phenolic acid (TPC) and antioxidant activity. High-performance liquid chromatography (HPLC) analysis identified 15 common components, including key marker compounds pinocembrin, 3-*O*-acetylpinobanksin, galangin, chrysin, benzyl *p*-coumarate, pinobanksin and caffeic acid phenethyl ester (CAPE). Cluster analysis (CA) and correlation coefficients (CC) analysis showed that these propolis could be divided into three distinct groups. Principal component analysis (PCA) and multiple linear regression analysis (MLRA) revealed that the contents of isoferulic acid, caffeic acid, CAPE, 3,4-dimethoxycinnamic acid, chrysin and apigenin are closely related to the antioxidant properties of propolis. In addition, eight peak areas decreased after reacting with 1,1-Diphenyl-2-picrylhydrazyl (DPPH) radicals, indicating that these compounds have antioxidant activity. The results indicate that the grouping and spectrum–effect relationship of Chinese propolis are related to their chemical compositions, and several compounds may serve as a better marker for the antioxidant activity of Chinese propolis than TFC and TPC. The findings may help to develop better methods to evaluate the quality of propolis from different geographic origins.

## 1. Introduction

Propolis is a biologically active natural product produced by honeybees collecting substances from parts of plants, buds and exudates [1]. Bees use propolis to build and repair their hives, such as for controlling the size of the hive door and repairing any cracks [2].

Propolis has a complex composition, with more than 500 compounds having been identified within it [3,4]. Many factors such as plant origin, geographic location and seasonality can influence the chemical composition of propolis by affecting plant bud exudates [5,6,7]. It is known that bees collect resin from more than 16 plant families, particularly the *Populus* family, with at least seven different *Populus* species having proven to be plant sources of propolis [6]. Previous studies indicate that the main plant sources of Chinese propolis are *Populus* species [8,9]. However, there are few studies on the classification of Chinese propolis to find different propolis types in China.

Propolis has demonstrated various bioactivities, and been used as a health supplement and food additive [10,11,12]. Among them, the antioxidant activity of propolis may play a key role in protection against the damage caused by free radicals in some chronic diseases [13]. The antioxidant activity of Chinese propolis is largely attributed to the high levels of various phenolic compounds, such as flavonoids and phenolic acids [14]. In this regard, analysis of the chemical composition and its relationship to biological activity, or the spectrum–effect relationship, is important to evaluate the quality of natural products [15,16,17].

Thus, we set out to investigate the spectrum–effect relationship of Chinese propolis by using high-performance liquid chromatography (HPLC) to separate and identify the chemical composition in 49 Chinese propolis samples collected from different regions. Furthermore, the total phenolic acid content (TPC), total flavonoid content (TFC) and antioxidant capacity of these propolis samples were determined. Chromatographic data were processed by multivariate analyses such as cluster analysis (CA), principal component analysis (PCA) and multi-linear regression analysis (MLRA), in order to classify samples and obtain the relationship between spectral and antioxidant capacity. The off-line anti-1,1-Diphenyl-2-picrylhydrazyl (DPPH) assay was performed to identify the antioxidant compounds in Chinese propolis.

## 2. Results and Discussion

### 2.1. HPLC Analysis of 49 Chinese Propolis

**Figure 1:**
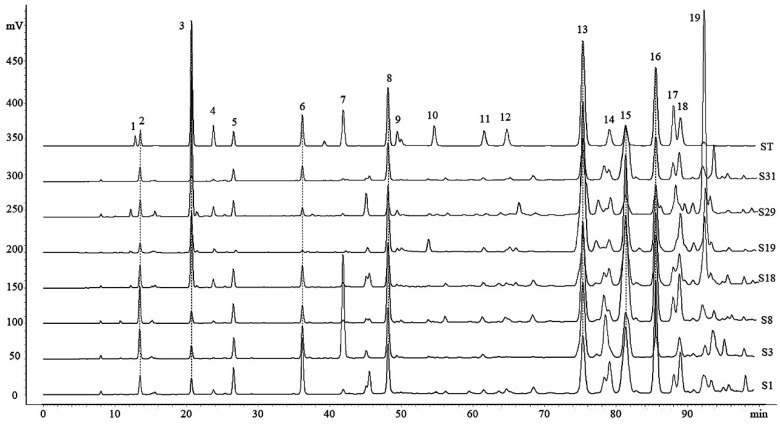
High-performance liquid chromatography (HPLC) chromatograms of the standard solution (ST) and Chinese propolis (S1,S3, S8, S18, S19, S29, S31): 1. Vanillic; 2. Caffeic acid; 3. *p*-Coumaric acid; 4. Ferulic acid; 5. Isoferulic acid; 6. 3,4-Dimethoxycinnamic acid; 7. Cinnamic acid; 8. Pinobanksin; 9. Naringenin; 10. Quercetin; 11. Kaempferol; 12. Apigenin; 13. Pinocembrin; 14. Benzyl caffeate; 15. 3-*O*-acetylpinobanksin; 16. Chrysin; 17. Caffeic acid phenethyl ester (CAPE); 18. Galangin; and 19. Benzyl *p*-coumarate.

The chromatographic profiles of 49 Chinese propolis samples (as detailed in Table 1) were analyzed using the previously established method [8,18,19]. The results of precision showed that the relative standard deviation (RSD) of the intraday and interday for retention times were less than 0.38% and 0.44%, respectively, and for peak areas were less than 2.54% and 2.69%. The RSD for the repeatability of retention time and peak areas were less than 0.31% and 5.71% (Table S1), respectively. The HPLC fingerprints of representative samples are shown in Figure 1, and the fingerprints of all samples are shown in Figure S1. 15 common peaks were identified by comparison with standard compound retention times, and the content of those compounds was quantified by the regression equation of standard compounds (Table S2). The contents of those compounds varied significantly with geographic origins (Table S3). The compounds with relative higher contents include pinocembrin (ranging from 20.14 to 104.90 mg/g, mean value 41.93 mg/g), 3-*O*-acetylpinobanksin (ranging from 3.26 to 73.08 mg/g, mean value 35.69 mg/g), galangin (ranging from 10.59 to 52.58 mg/g, mean value 33.45 mg/g), chrysin (ranging from 5.26 to 52.91 mg/g, mean value 33.12 mg/g), benzyl *p*-coumarate (ranging from 5.04 to 121.39 mg/g, mean value 27.98 mg/g), pinobanksin (ranging from 2.27 to 51.27 mg/g, mean value 23.51 mg/g) and caffeic acid phenethyl ester (CAPE) (ranging from 0 to 49.58 mg/g, mean value 12.26 mg/g). The chemical composition of propolis in all regions showed a similar characteristic as the poplar-type propolis [20]. This may be related to the fact that *Populus* is widespread throughout China [21,22], and research shows that *Apis mellifera* prefer *Populus* as plant sources [6]. The content of common compounds varies between different samples, as the chemical compositions of propolis could be influenced by botanical origin, collecting season or other factors [23].

### 2.2. Similarity of HPLC Fingerprints among 49 Chinese Propolis

The similarity of 49 propolis fingerprints was analyzed using the software “Similarity Evaluation System for Chromatographic Fingerprint of Tradition Chinese Medicine (TCM)” developed by the Chinese Pharmacopoeia Commission. The results showed that the correlation coefficients (CC) values of most Chinese propolis fingerprints were higher than 0.6, indicating that their compositions were very similar. However, the CC values of five samples (S21, 0.512; S23, 0.306; S25, 0.356; S27, 0.407; and S29, 0.499) collected from Northeast China were significantly lower than that of other propolis, indicating the difference of these samples with other Chinese propolis (Table 1).

**Figure 2:**
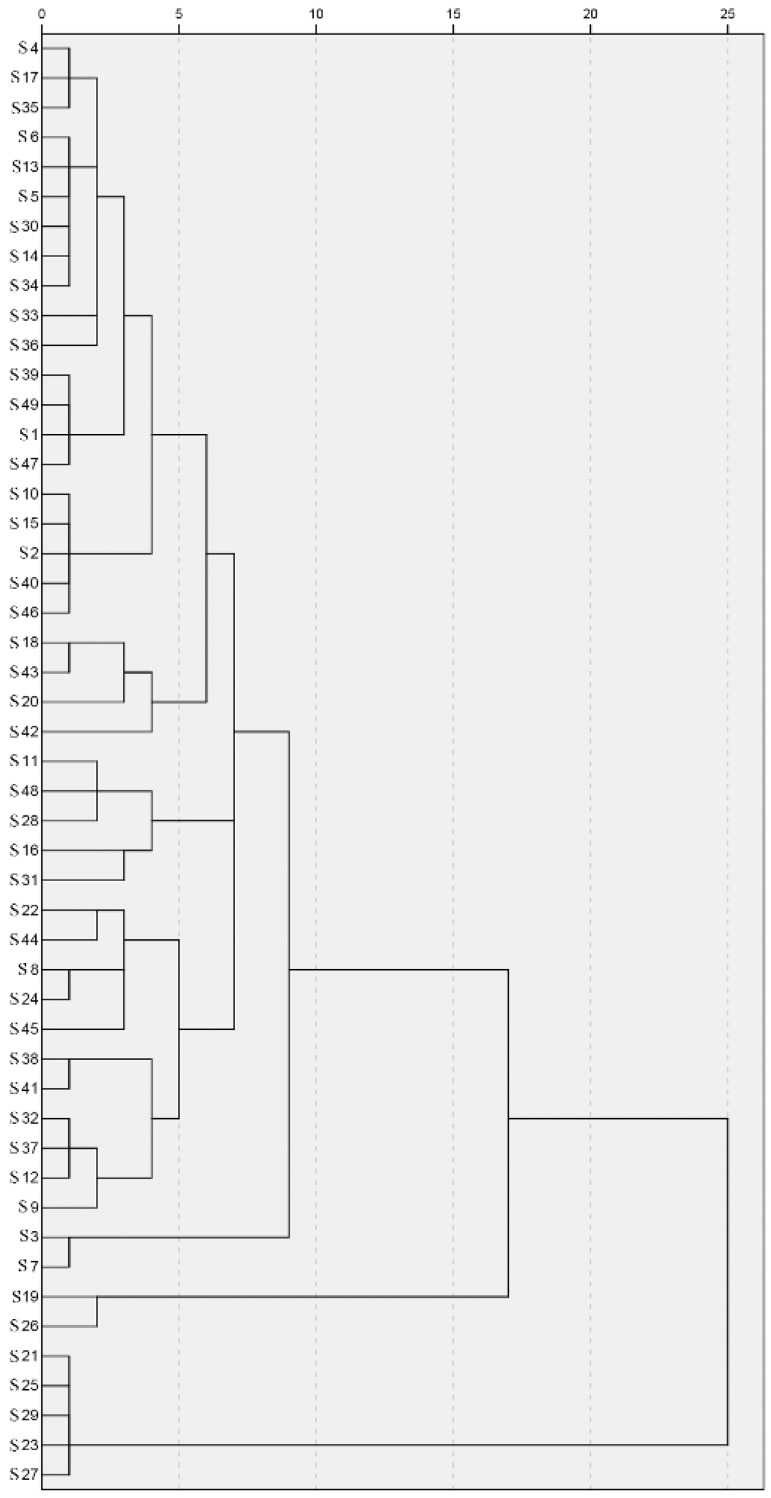
Dendrogram of cluster analysis of 49 Chinese propolis samples (S1–S49).

The CA identified three distinctive groups: Group 1 contains 42 propolis samples; Group 2 contains S19 and S26; and Group 3 contains S21, S25, S29, S23 and S27 (Figure 2). All samples in Groups 2 and 3 were collected in Northeast China, which contained rich *p*-coumaric acid and benzyl *p*-coumarate. This is consistent with the results of the CC values; the CC values of all samples in Group 3 are below 0.5.

These results indicate that some propolis from Northeast China was special, and could be classified as a new type of propolis. We have previously shown that the propolis collected from the Changbai Mountain area (CBM) in Northeast China had a higher content of *p*-coumaric acid and benzyl *p*-coumarate [18]. Accordingly, samples in Group 1 were ordinary Chinese propolis, samples in Group 2 were mixed propolis and samples in Group 3 were CBM propolis. Samples in the same group may also be subdivided into different subgroups, but further research is needed to establish this. As mentioned earlier, many factors can affect the chemical composition of propolis [5,6]. Chinese propolis could be divided into different groups, which may be caused by differences in plant sources, climate and other factors in different regions.

### 2.3. Contents of Flavonoids and Phenolics of 49 Chinese Propolis

It has been well established that flavonoids and phenolic acids, as the secondary metabolites in plants with broad biological activities [24,25], are the main active substances in poplar-type propolis [26]. TFC and TPC have been widely used as indicators for evaluating the quality of propolis. Table 2 shows the results of TFC and TPC of 49 propolis samples, determined by the methods described previously [27]. There is a significant variation of TFC and TPC among these samples, ranging from 63.75 ± 1.92 mg/g to 454.92 ± 32.67 mg/g for TFC and 150.83 ± 2.75 mg/g to 556.3 ± 5.55 mg/g for TPC, respectively. These results are consistent with the previous report [28]. The variations of TFC and TPC in these samples are in line with the chemical variations as shown above, indicating that the differences in these propolis samples are related to their geographic origins.

### 2.4. DPPH Scavenging Activity of 49 Chinese Propolis

DPPH assay has been widely used as a sensitive method to assess the antioxidant capacity of various samples [29]. We determined the DPPH scavenging activity (IC_50_) of these Chinese propolis samples. As shown in Table 2, all propolis samples showed strong antioxidant activity. The IC_50_ values of these propolis samples varied widely, ranging from 71.19 ± 5.31 μg/mL to 432.08 ± 6.42 μg/mL, indicating that the antioxidant activity of Chinese propolis is also region-dependent. In addition, there was a significant negative correlation between DPPH scavenging activity (IC_50_) and TPC (R = −0.469, *p* < 0.01), but not TFC (R = −0.260, *p* > 0.05). These results indicate that the phenolic acids have a greater influence on the propolis antioxidant capacity than the flavonoids, which was consistent with previous research [27,30]. The antioxidative activity of propolis is the most appreciated property, and a variety of biological activities of propolis largely results from their antioxidative effects [31,32]. Therefore, the antioxidant capacity is an important indicator of the quality of propolis.

### 2.5. Spectrum–Effect Relationship of 49 Chinese Propolis

**Figure 3:**
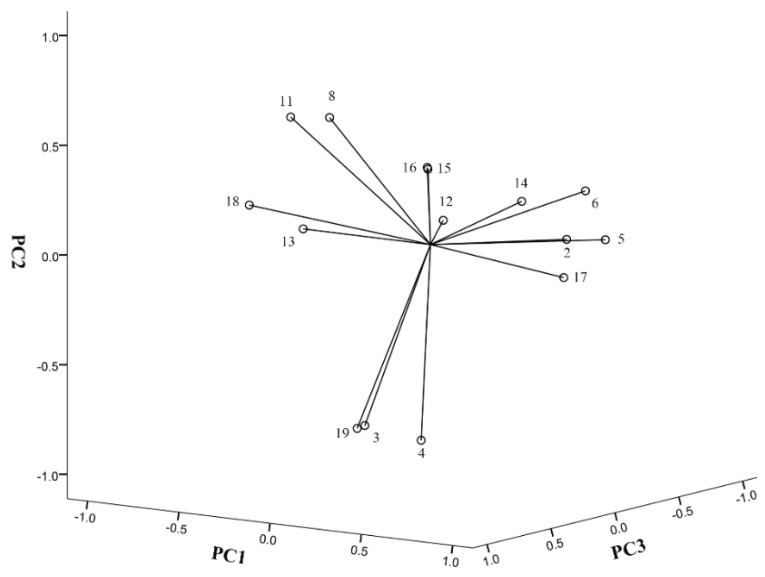
The loadings plots of the principal components. See Table 3 for compound names.

MLRA is a useful method to quantify the relationship between spectrum and bioactivities [33]. However, when independent variables have collinear relationships, the MLRA model is unreliable. PCA could reduce the dimensionality of data and convert correlated data into a few integrated variables without collinearity [34,35]. In this study, we firstly conducted PCA and identified four principal components (PC) which contained 79.99% information of the original data (PC1, 35.44%; PC2, 25.81%; PC3, 10.10%; and PC4, 8.64%). As shown in Table 3 and Figure 3, the PC1 was highly positively correlated to the contents of isoferulic acid, caffeic acid, CAPE and 3,4-dimethoxycinnamic acid. The PC2 was positively correlated to the contents of kaempferol, but negatively correlated to the contents of ferulic acid benzyl *p*-coumarate and *p*-coumaric acid. The PC3 was positively correlated to the contents of galangin, and negatively correlated to benzyl caffeate content. The PC4 was positively correlated to the contents of chrysin and apigenin. Based on PCA results, we further performed MLRA and found that PC1 and PC4 had a greater impact on the antioxidant capacity, while the impacts of PC2 and PC3 were less significant. The equations were: IC_50_ = 169.795-68.899PC1-23.475PC4. These findings indicate that isoferulic acid, caffeic acid, CAPE and 3,4-dimethoxycinnamic acid in PC1 and chrysin and apigenin in PC4 contribute more to the antioxidant capacity of Chinese propolis. The total content of these compounds showed a superior negative correlation with the DPPH scavenging activity (IC_50_) (R = −0.716, *p <* 0.001). Thus, the content of these compounds could be used as an indicator to predict the antioxidant capacity of propolis. However, this does not mean that all these compounds have antioxidant activity. Conversely, the antioxidant compounds in propolis may not only be these compounds. In addition to MLRA and PCA, there are many other types of statistical analysis methods on spectrum–efficacy studies, and different methods have different emphases [36,37,38].

### 2.6. Determination of Antioxidant Compounds in Propolis by Off-Line Anti-DPPH Assay

**Figure 4:**
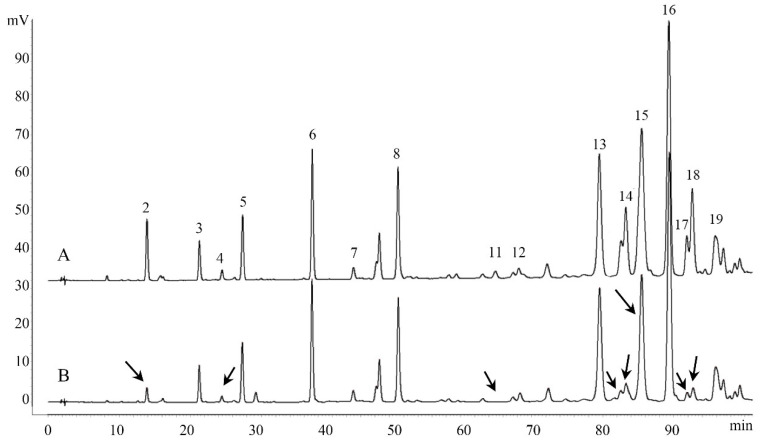
HPLC chromatograms of the Chinese propolis before (**A**) and after (**B**) reacting with DPPH radical. Arrows indicate peaks with a decreasing peak area.

To directly determine the nature of antioxidant compounds in Chinese propolis, an off-line anti-DPPH assay was performed. Eight compounds (caffeic acid, ferulic acid, kaempferol, unknown compound 1, benzyl caffeate, 3-*O*-acetylpinobanksin, CAPE and galangin) were found with a decrease in peak area after the reaction, indicating that these compounds have antioxidant activity (Figure 4). Other studies on the antioxidant capacity of Chinese propolis have also confirmed that these compounds have the DPPH scavenging activity [14,39,40]. Furthermore, in the related research about Brazil green propolis, nine compounds, including caffeic acid and kaempferol, show a decrease in peak area [27]. Unlike the ambiguity and integrity of the spectrum–effect relationship, this method could detect antioxidant compounds in propolis, as well as in other natural products. In addition, there is an on-line anti-DPPH assay similar to this method, which can monitor the reaction in real time, but requires an additional post-column system [41].

## 3. Materials and Methods

### 3.1. Chemicals and Reagents

HPLC-grade methanol was purchased from Merck (Merck & Co., Inc., Billerica, MA, USA), and analytical grade acetic acid and absolute ethanol were purchased from Chemical Reagent Factory of Zhejiang University (Hangzhou, Zhejiang, China). Absolute alcohol and acetic acid were purchased from Shanghai Chemical Reagent Company of Chinese Medical Group (Shanghai, China). Ultra-Pure water was purified by the Yjd-upws Ultra-Pure water system (Hangzhou, Zhejiang, China).

DPPH, vanillic acid, caffeic acid, ferulic acid, isoferulic acid, *p*-coumaric acid, cinnamic acid, 3,4-dimethoxycinnamic acid, CAPE, myricetin, apigenin, galangin, chrysin, pinocembrin, quercetin, kaempferol, luteolin and naringenin were purchased from Sigma–Aldrich (St. Louis, MI, USA), pinobanksin and 3-*O*-acetylpinobanksin were purchased from Ningbo Haishu Apexocean Biochemicals Co., Ltd (Ningbo, Zhejiang, China), and benzyl *p*-coumarate was purchased from Kunming BioBioPha Co., Ltd (Kunming, Yunnan, China).

### 3.2. Samples Collection and Preparation

49 propolis samples (S01–S49) used in this study were harvested by scratching from beehives in 49 cities of 16 provinces (Table 1). The propolis samples sites cover the main Apis mellifera breeding areas and propolis production areas in China.

The frozen propolis samples were extracted, as reported previously [8]. The raw propolis samples (3.0 g) were extracted with 50 mL of a 95% hydro-alcoholic solution in an ultrasonic water bath for 45 min. The mixture was then centrifuged, and the sediment was re-extracted twice under the same conditions. The supernatant was kept in a refrigerator overnight and filtered to remove impurities. After that, the filtered solution was evaporated to dryness. The dry residue powder of propolis (0.2 g) was then redissolved in 10 mL ethanol (20 mg/mL).

### 3.3. HPLC Procedures

Chromatographic analysis was performed with Agilent 1200 Series (Agilent Technologies Inc., Santa Clara, CA, USA) equipment. Separation was achieved on a Sepax HP-C18 column (150 × 4.6 mm, 5 μm; Sepax Technologies Inc., Newark, DE, USA) and maintained at 33 °C. The mobile phase was maintained at a constant flow rate of 1 mL/min. The gradient elution, which consisted of aqueous phase A (1% acetic acid) and organic phase B (anhydrous methanol), was adjusted as we previously reported, in detail: 15% to 35% (B) 0 to 30 min; 35% to 44% (B) 30 to 46 min; 44% to 50% (B) 46 to 70 min; 50% to 52% (B) 70 to 77 min; 52% to 60% (B) 77 to 92 min; 60% to 75% (B) 92 to 115 min; 75% to 100% (B) 115 to 125 min; and 100% to 15% (B) 125 to 135 min [18]. Each sample (5 μL) was purified with 0.45 μm filters, and then injected through an automatic sampler system and monitored by a UV detector at 280 nm.

The methodology was validated through intraday precision, intraday precision and repeatability tests. The contents of identified compounds in propolis were quantified using the respective regression equation of standard substances. The peaks-area was quantitated by external calibration, the standards compounds were dissolved in methanol, the mixed standard solution was prepared and a series of working standard solutions were prepared according to the level of these reference standards expected in samples.

### 3.4. Determinations of Total Flavonoids and Total Phenolics

TFC was determined using the method reported previously, with minor modifications [27,42]. Briefly, 60 μL of propolis ethanol solution (0.2 mg/mL) was mixed with 40 μL (100 g/L) aluminum nitrate and 40 μL (9.8 g/L) potassium acetate, and adjusted to 200 μL with distilled water. The mixed solution was kept in a dark room for 1 h at room temperature, and then measured the absorbance at 415 nm using a microplate reader (Bio-Rad, Hercules, CA, USA).

TPC was measured by the Folin–Ciocalteau method [27,43]. Briefly, a 100 μL Folin–Ciocalteau reagent was added to 100 μL of propolis ethanol solution (10 mg/mL), and then mixed with 500 μL (1 mol/L) sodium carbonate and adjusted to 1 mL with distilled water. The mixed solution was kept in a dark room for 1 h at room temperature, and then measured the absorbance at 760 nm using the microplate reader.

### 3.5. Antioxidant Capacity

Fresh DPPH stock solution was prepared by dissolving 30 mg DPPH in 10 mL ethanol (3 mg/mL). The DPPH scavenging activity was determined according to the method reported previously, with modifications [27,44]. In brief, 100 μL DPPH working solution was mixed with 100 μL propolis extract in a 96-well plate, and incubated for 30 min in the dark. The absorbance of the reaction solutions was then measured at 517 nm using the microplate reader. The results were expressed as IC_50_ (μg/mL, the concentration of scavenging 50% DPPH radical).

### 3.6. Off-line Anti-DPPH Assay

The off-line anti-DHHP assay was based on the method reported previously, with some modifications [27,45]. Briefly, 10 mg/mL DPPH ethanol solution was mixed with an equal volume of 5 mg/mL Chinese propolis ethanol solution, and then placed in a dark room for 30 min. After filtration, the mixed solution was determined by HPLC, using the procedures as described above.

### 3.7. Statistical Analysis and Chemometric Application

The software “Similarity Evaluation System for Chromatographic Fingerprint of TCM”, published by the Chinese Pharmacopoeia Commission (Chinese Pharmacopoeia Commission, Version 2012.130723), was used to synchronize and conduct qualitative and quantitative comparisons for all propolis samples. The reference fingerprint was formed by the system using the median method from the chromatograms of 49 propolis, and the similarity values of each propolis extract and reference fingerprint were also determined. The means and standard deviations (SD) were calculated using the Microsoft Excel 2016 software (Microsoft Inc., Redmond, WA, USA). The CA, HCA, MLRA and IC_50_ of 49 propolis were performed using SPSS 22 statistics software (SPSS Inc., Armonk, NY, USA).

## 4. Conclusion

In this study, 49 propolis samples collected from different regions in China were studied for their chemical profiles, antioxidant activity and spectrum–effect relationship. The results showed that the Chinese propolis could be divided into three different types according to the similarity of their HPLC fingerprints, with propolis collected from Changbai Mountain in Northeast China, which contained a higher content of *p*-coumaric acid and benzyl *p*-coumarate as a distinct type. The spectrum–effect relationship showed that the contents of isoferulic acid, caffeic acid, CAPE, 3,4-dimethoxycinnamic acid, chrysin and apigenin in Chinese propolis could be related to the antioxidant activity of propolis samples. Furthermore, eight active compounds were identified with anti-DPPH activities in Chinese propolis. The results indicate that the grouping and spectrum–effect relationship of Chinese propolis are related to their chemical compositions, and several compounds may serve as a better marker for the antioxidant activity of Chinese propolis than TFC and TPC. The findings may help to develop better methods to evaluate the quality of propolis from different geographic origins.

## Figures and Tables

**Table 1:** Collection site correlation coefficients of 49 Chinese propolis.

Samples No.	Collected Site (city, province)	Correlation Coefficients	Samples No.	Collected Site (City, Province)	Correlation Coefficients
S1	Lujiang, Anhui	0.891	S26	Yichun, Heilongjiang	0.822
S2	Tongcheng, Anhui	0.861	S27	Yilan, Heilongjiang	0.407
S3	Shouxian, Anhui	0.675	S28	Baoqing, Heilongjiang	0.892
S4	Mingcong, Anhui	0.896	S29	Muling, Heilongjiang	0.499
S5	Huaibei, Anhui	0.887	S30	Xinye, Henan	0.924
S6	Fuyang, Anhui	0.914	S31	Shangqiu, Henan	0.881
S7	Mengcheng, Guizhou	0.533	S32	Fangcheng, Henan	0.915
S8	Meishan, Sichuan	0.926	S33	Shangshui, Henan	0.861
S9	Xicuan, Sichuan	0.890	S34	Nanyang, Henan	0.889
S10	Qixia, Shandong	0.880	S35	Huaibin, Henan	0.875
S11	Dezhou, Shandong	0.825	S36	Liutai, Jiangsu	0.894
S12	Heze, Shandong	0.878	S37	Jianhu, Jiangsu	0.901
S13	Longkou, Shandong	0.921	S38	Yicheng, Hubei	0.818
S14	Donge, Shandong	0.890	S39	Gucheng, Hubei	0.895
S15	Penglai, Shandong	0.879	S40	Dongkou, Hubei 1	0.796
S16	Yanbailou, Shaanxi	0.811	S41	Dongkou, Hubei 2	0.789
S17	Mizhi, Shaanxi	0.869	S42	Xianju, Zhejiang	0.767
S18	Fusong, Jilin	0.906	S43	Jinyun, Zhejiang	0.829
S19	Baishan, Jilin	0.846	S44	Penghu, Ningxia	0.889
S20	Jian, Jilin	0.848	S45	Gongliu, Xinjiang	0.836
S21	Dashiqiao, Liaoning	0.512	S46	Beijing	0.826
S22	Faku, Liaoning	0.930	S47	Baotou, Inner Mongolia	0.926
S23	Suizhong, Liaoning	0.306	S48	Huailai, Hebei	0.687
S24	Shuangyashan, Heilongjiang	0.921	S49	Qiuxian, Hebei	0.910
S25	Gannan, Heilongjiang	0.356			

**Table 2:** Total phenolic acid (TPC) contents, contents of total flavonoids (TFC) and 1,1-Diphenyl-2-picrylhydrazyl (DPPH) scavenging activity of 49 Chinese propolis.

Samples No.	TPC (mg/g, GAE)	TFC (mg/g, QE)	DPPH Scavenging Activity (IC_50_,μg/mL)	Samples No.	TPC (mg/g, GAE)	TFC (mg/g, QE)	DPPH Scavenging Activity (IC_50_,μg/mL)
S1	404.47 ± 10.72	361.59 ± 8.54	90.51 ± 0.89	S26	232.59 ± 3.18	146.63 ± 3.33	294.04 ± 10.26
S2	249.95 ± 4.52	162.39 ± 13.93	113.19 ± 5.93	S27	199.70 ± 3.83	75.43 ± 4.19	216.06 ± 3.30
S3	223.12 ± 1.69	110.56 ± 1.24	101.21 ± 1.87	S28	275.54 ± 2.91	167.16 ± 6.34	83.47 ± 3.55
S4	248.62 ± 2.22	135.56 ± 4.49	122.88 ± 1.73	S29	208.31 ± 2.45	77.76 ± 4.67	171.61 ± 8.54
S5	529.49 ± 4.10	427.56 ± 17.03	124.35 ± 1.36	S30	231.11 ± 2.03	141.17 ± 2.84	112.14 ± 5.71
S6	556.3 ± 5.55	454.92 ± 32.67	113.10 ± 4.67	S31	170.67 ± 0.94	106.06 ± 1.34	171.52 ± 8.21
S7	236.4 ± 3.17	102.50 ± 1.17	92.45 ± 8.31	S32	219.01 ± 2.30	204.76 ± 4.05	233.6 ± 10.59
S8	257.15 ± 3.76	191.19 ± 1.55	104.17 ± 6.69	S33	232.35 ± 5.43	210.53 ± 4.05	153.93 ± 3.81
S9	236.92 ± 6.38	205.32 ± 6.12	164.27 ± 7.47	S34	242.33 ± 1.11	149.68 ± 2.55	119.5 ± 4.51
S10	276.21 ± 4.99	190.72 ± 12.24	108.17 ± 5.66	S35	228.94 ± 2.76	192.23 ± 6.62	165.23 ± 4.23
S11	272.91 ± 3.51	186.96 ± 5.35	73.56 ± 2.61	S36	223.90 ± 2.22	216.31 ± 4.29	241.85 ± 11.63
S12	210.23 ± 4.14	205.95 ± 1.68	308.11 ± 6.36	S37	228.39 ± 2.45	223.83 ± 1.15	269.08 ± 1.94
S13	242.81 ± 2.60	197.64 ± 2.02	124.54 ± 5.94	S38	197.97 ± 1.84	203.46 ± 6.06	303.87 ± 6.00
S14	237.32 ± 3.06	179.05 ± 7.48	124.16 ± 9.21	S39	252.31 ± 5.44	167.96 ± 4.77	100.20 ± 1.46
S15	246.23 ± 5.42	159.25 ± 1.50	109.00 ± 3.77	S40	231.12 ± 4.63	143.61 ± 5.02	132.98 ± 3.55
S16	254.92 ± 5.02	143.97 ± 3.06	76.06 ± 3.15	S41	205.82 ± 5.02	208.93 ± 3.23	404.56 ± 11.9
S17	216.69 ± 2.12	158.80 ± 3.48	133.82 ± 2.93	S42	150.83 ± 2.75	70.08 ± 4.15	354.31 ± 5.12
S18	256.96 ± 3.37	163.24 ± 5.62	122.64 ± 9.30	S43	234.55 ± 5.82	126.88 ± 7.89	146.45 ± 6.61
S19	217.37 ± 3.40	133.04 ± 4.02	352.75 ± 9.75	S44	268.44 ± 2.77	194.49 ± 4.98	124.45 ± 6.19
S20	139.92 ± 1.90	63.75 ± 1.92	432.08 ± 6.42	S45	312.01 ± 4.34	207.96 ± 9.33	132.46 ± 4.53
S21	223.74 ± 1.30	100.90 ± 0.42	197.34 ± 6.78	S46	252.51 ± 1.40	149.41 ± 9.75	91.01 ± 9.85
S22	302.55 ± 6.12	219.2 ± 2.56	87.14 ± 7.38	S47	251.63 ± 0.80	172.76 ± 9.53	125.20 ± 5.37
S23	178.37 ± 0.89	53.45 ± 3.41	277.77 ± 10.99	S48	274.21 ± 2.41	134.62 ± 3.64	71.19 ± 5.31
S24	240.99 ± 4.03	171.38 ± 6.69	124.92 ± 6.32	S49	247.34 ± 4.43	173.99 ± 7.50	113.08 ± 6.15
S25	188.86 ± 1.80	73.51 ± 9.97	309.97 ± 10.23				

Data are shown as the mean ± SD (*n* = 3). GAE, gallic acid equivalent; QE, quercetin equivalent.

**Table 3:** The loadings of the first four rotated principal components.

The Loading					
Peak No.	Compound	PC1 (35.44%)	PC2 (25.81%)	PC3 (10.10%)	PC4 (8.64%)
5	Isoferulic acid	**0.911**	0.083	−0.211	0.064
2	Caffeic acid	**0.869**	0.115	0.032	0.015
17	CAPE	**0.825**	−0.069	−0.009	0.312
6	3,4-Dimethoxycinnamic acid	**0.745**	0.277	−0.292	0.236
4	Ferulic acid	−0.034	**−0.914**	−0.119	0.087
19	Benzyl *p*-coumarate	−0.238	**−0.850**	0.090	−0.357
3	*p*-Coumaric acid	−0.249	**−0.849**	0.015	−0.354
11	Kaempferol	−0.041	**0.659**	0.587	0.169
13	Pinobanksin	−0.386	0.599	0.398	0.427
14	Benzyl caffeate	0.063	0.092	**−0.769**	−0.024
18	Galangin	−0.396	0.242	**0.709**	0.370
15	3-*O*-acetylpinobanksin	0.496	0.486	0.589	0.015
8	Pinocembrin	−0.218	0.127	0.541	−0.495
16	Chrysin	0.103	0.356	0.023	**0.829**
12	Apigenin	0.309	0.170	0.198	**0.757**

Bold fonts indicate high score (absolute value greater than 0.6).

## Supplementary Materials

The following are available online at https://www.mdpi.com/1420-3049/25/14/3243/s1, Table S1. Precision and repeatability data of 15 compounds in Chinese propolis. Table S2: The regression equation of standard compounds in Chinese propolis. Table S3: The content of common compounds in different Chinese propolis. Figure S1: HPLC chromatograms of the standard solution and Chinese propolis.
