# Supplementary material for: Grouping, Spectrum–Effect Relationship and Antioxidant Compounds of Chinese Propolis from Different Regions Using Multivariate Analyses and Off-Line Anti-DPPH Assay
Source: Molecules. 2020 Jul 16;25(14):3243. doi: 10.3390/molecules25143243 (PMC7397058; doi:10.3390/molecules25143243)
Supplement: Supplementary file 1 [file molecules-25-03243-s001.zip › new folder/Figure 1S.docx]

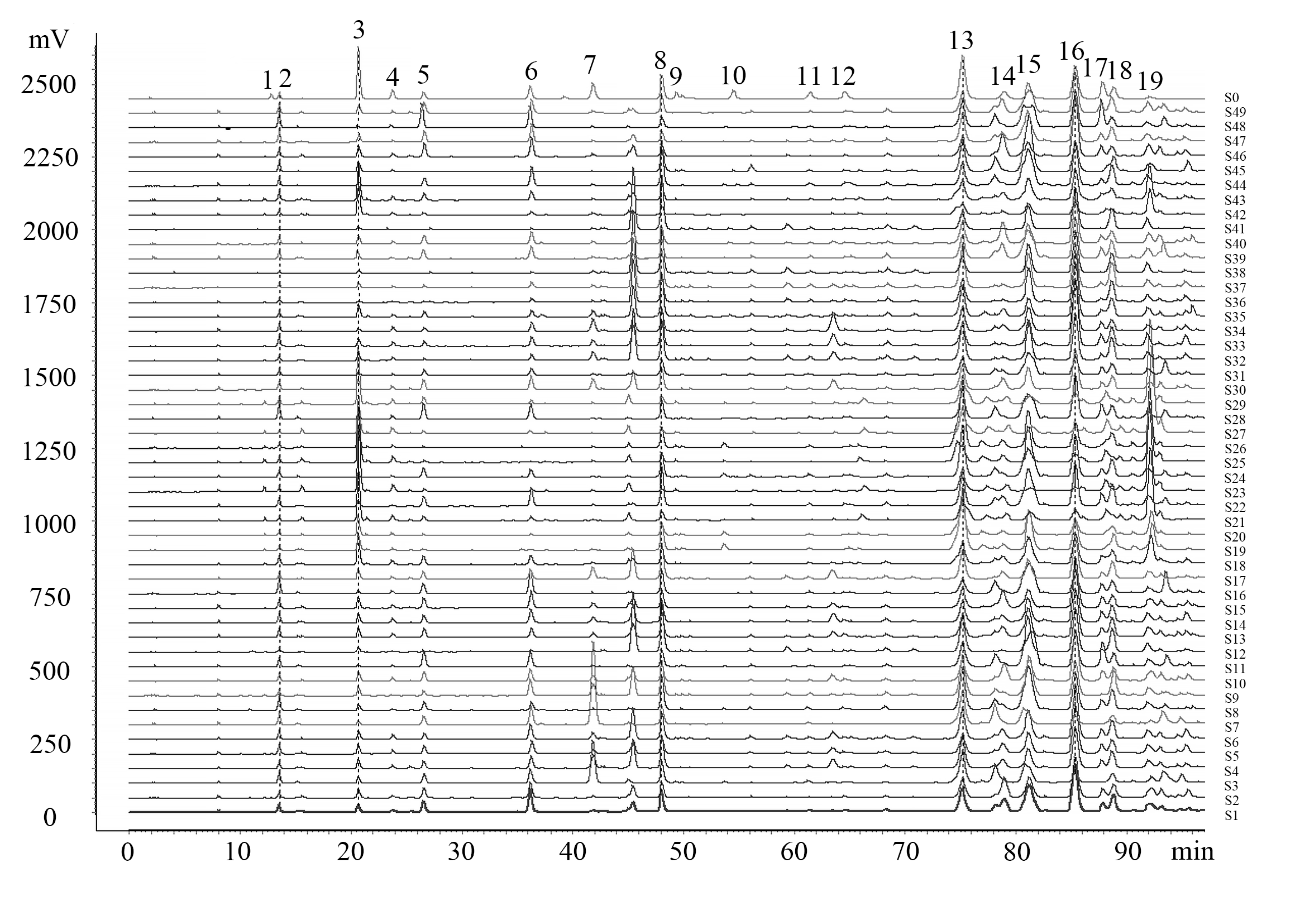


**Figure 1S.** HPLC chromatograms of the standard solution (S0) and Chinese propolis (S1-S49): 1. Vanillic; 2. Caffeic acid; 3. *p*-Coumaric acid; 4. Ferulic acid; 5. Isoferulic acid; 6. 3,4-Dimethoxycinnamic acid; 7. Cinnamic acid; 8. Pinobanksin; 9. Naringenin;10. Quercetin; 11. Kaempferol; 12. Apigenin; 13. Pinocembrin; 14. Benzyl caffeate;15. Pinobanksin 3-oacetate; 16. Chrysin; 17. CAPE; 18. Galangin; 19. Benzyl *p*-coumarate.
