# Supplementary material for: Grouping, Spectrum–Effect Relationship and Antioxidant Compounds of Chinese Propolis from Different Regions Using Multivariate Analyses and Off-Line Anti-DPPH Assay
Source: Molecules. 2020 Jul 16;25(14):3243. doi: 10.3390/molecules25143243 (PMC7397058; doi:10.3390/molecules25143243)
Supplement: Supplementary file 1 [file molecules-25-03243-s001.zip › new folder/Table 1S.docx]

**Table 1S.** Precision and repeatability data of 15 compounds in Chinese propolis.

| Compound | intraday precision (RSD%, n=6) | | intraday precision (RSD%, n=6) | | Repeatability (RSD%, n=6) | |
| --- | --- | --- | --- | --- | --- | --- |
|  | Retention time | Peak area | Retention time | Peak area | Retention time | Peak area |
| Caffeic acid | 0.35 | 1.12 | 0.32 | 1.37 | 0.29 | 3.30 |
| *p*-Coumaric acid | 0.24 | 0.98 | 0.31 | 1.38 | 0.12 | 2.21 |
| Ferulic acid | 0.36 | 2.02 | 0.24 | 1.98 | 0.17 | 4.21 |
| Isoferulic acid | 0.22 | 1.35 | 0.19 | 2.07 | 0.22 | 3.42 |
| 3,4-Dimethoxycinnamic acid | 0.12 | 1.89 | 0.29 | 2.45 | 0.31 | 2.87 |
| Pinobanksin | 0.33 | 0.87 | 0.21 | 1.22 | 0.23 | 2.12 |
| Kaempferol | 0.21 | 2.43 | 0.44 | 2.69 | 0.11 | 3.23 |
| Apigenin | 0.22 | 1.49 | 0.28 | 1.89 | 0.25 | 4.21 |
| Pinocembrin | 0.31 | 1.78 | 0.36 | 2.16 | 0.30 | 1.12 |
| Benzyl caffeate | 0.38 | 1.23 | 0.21 | 1.54 | 0.17 | 2.05 |
| 3-O-acetylpinobanksin | 0.38 | 2.54 | 0.22 | 2.31 | 0.16 | 1.90 |
| Chrysin | 0.13 | 1.67 | 0.32 | 1.98 | 0.24 | 1.65 |
| CAPE | 0.22 | 1.21 | 0.31 | 2.30 | 0.18 | 2.43 |
| Galangin | 0.27 | 1.56 | 0.29 | 1.81 | 0.22 | 5.71 |
| Benzyl *p*-coumarate | 0.19 | 1.86 | 0.27 | 1.72 | 0.21 | 3.27 |
