# Supplementary material for: Grouping, Spectrum–Effect Relationship and Antioxidant Compounds of Chinese Propolis from Different Regions Using Multivariate Analyses and Off-Line Anti-DPPH Assay
Source: Molecules. 2020 Jul 16;25(14):3243. doi: 10.3390/molecules25143243 (PMC7397058; doi:10.3390/molecules25143243)
Supplement: Supplementary file 1 [file molecules-25-03243-s001.zip › new folder/Table 2S.docx]

**Table 2S.** The regression equation of standard compounds in Chinese propolis.

| Compound | Regression equation | R² |
| --- | --- | --- |
| Caffeic acid | y = 19.961x - 4.644 | R² = 0.9974 |
| *p*-Coumaric acid | y = 18.821x - 9.5091 | R² = 0.9976 |
| Ferulic acid | y = 14.423x - 0.2291 | R² = 0.9975 |
| Isoferulic acid | y = 14.930x + 3.201 | R² = 0.9969 |
| 3,4-Dimethoxycinnamic acid | y = 14.644x + 4.468 | R² = 0.9967 |
| Pinobanksin | y = 15.701x - 2.196 | R² = 0.9963 |
| Kaempferol | y = 10.468x - 20.948 | R² = 0.9846 |
| Apigenin | y = 13.507x - 14.146 | R² = 0.9929 |
| Pinocembrin | y = 17.531x + 25.354 | R² = 0.9970 |
| Benzyl caffeate | y = 9.729x + 0.049 | R² = 0.9972 |
| 3-O-acetylpinobanksin | y = 23.389x + 14.159 | R² = 0.9969 |
| Chrysin | y = 24.474x - 28.395 | R² = 0.9977 |
| CAPE | y = 11.024x + 5.709 | R² = 0.9971 |
| Galangin | y = 10.560x - 35.330 | R² = 0.9980 |
| Benzyl *p*-coumarate | y = 14.182x + 11.489 | R² = 0.9999 |
