# Supplementary material for: Grouping, Spectrum–Effect Relationship and Antioxidant Compounds of Chinese Propolis from Different Regions Using Multivariate Analyses and Off-Line Anti-DPPH Assay
Source: Molecules. 2020 Jul 16;25(14):3243. doi: 10.3390/molecules25143243 (PMC7397058; doi:10.3390/molecules25143243)
Supplement: Supplementary file 1 [file molecules-25-03243-s001.zip › new folder/Table 3S.docx]

**Table 3S.** The content of common compounds in different Chinese propolis.

| Samples No. | Amount (mg/g) | | | | | | | | | | | | | | |
| --- | --- | --- | --- | --- | --- | --- | --- | --- | --- | --- | --- | --- | --- | --- | --- |
|  | Caffeic acid | *p*-Coumaric acid | Ferulic acid | Isoferulic acid | 3,4-Dimethoxycinnamic acid | Pinobanksin | Kaempferol | Apigenin | Pinocembrin | Benzyl caffeate | Pinobanksin 3-oacetyl | Chrysin | CAPE | Galangin | Benzyl *p*-coumarate |
| S1 | 4.67±0.16 | 4.41±0.07 | 1.65±0.06 | 9.44±0.01 | 21.63±0.21 | 21.19±0.11 | 3.56±0.05 | 4.55±0.25 | 35.44±0.12 | 34.21±1.03 | 41.92±0.06 | 43.59±0.37 | 13.73±0.03 | 36.90±0.10 | 17.41±0.02 |
| S2 | 3.52±0.24 | 4.83±0.22 | 1.92±0.04 | 7.8±0.02 | 13.9±0.02 | 15.47±0.04 | 1.96±0.01 | 2.45±0.01 | 28.39±0.07 | 57.37±3.42 | 25.77±0.19 | 38.61±0.31 | 13.67±0.13 | 28.75±0.07 | 17.21±0.06 |
| S3 | 7.23±0.41 | 3.57±0.13 | / | 7.28±0.01 | 12.52±0.23 | 21.43±0.34 | 3.07±0.03 | / | 48.78±0.14 | 51.6±0.91 | 30.46±0.11 | 8.48±0.08 | / | 18.58±0.03 | 7.17±0.12 |
| S4 | 3.99±0.08 | 5.57±0.01 | 2.23±0.08 | 5.39±0.01 | 11.33±0.12 | 34.3±0.03 | 3.89±0.02 | 2.03±0.03 | 44.40±0.51 | 20.45±0.41 | 22.18±0.06 | 37.94±0.54 | 12.12±0.15 | 35.19±0.16 | 12.22±0.04 |
| S5 | 5.91±0.16 | 5.55±0.09 | 2.8±0.05 | 6.43±0.04 | 12.09±0.04 | 17.02±0.06 | 2.02±0.01 | 3.23±0.01 | 27.37±0.02 | 25.43±1.53 | 24.25±0.03 | 45.41±0.34 | 15.73±0.13 | 28.7±0.09 | 14.28±0.05 |
| S6 | 5.66±0.11 | 4.47±0.09 | 3.33±0.01 | 4.35±0 | 13.24±0.01 | 27.02±0.03 | 3.34±0.01 | 2.54±0.16 | 43.90±6.34 | 23.68±0.39 | 35.94±0.03 | 50.22±0.25 | 16.09±0.05 | 39.54±0.08 | 13.87±0.05 |
| S7 | 7.48±0.20 | 2.2±0.06 | / | 6.94±0.01 | 16.65±1.03 | 19.17±0.17 | 3.18±0.01 | 0.21±0.25 | 49.84±2.01 | 54.51±0.03 | 25.05±0.13 | 6.86±0.03 | / | 18.74±0.02 | 5.04±0.12 |
| S8 | 9.29±0.31 | 3.36±0.08 | 0.79±0.02 | 6.71±0.01 | 6.46±0.02 | 34.00±0.02 | 4.49±0.02 | 5.11±0.02 | 59.69±2.11 | 10.39±1.28 | 70.07±0.22 | 34.18±0.12 | 18.43±0.11 | 42.95±0.09 | 13.69±0.03 |
| S9 | 3.35±0.81 | 5.43±0.04 | 0.53±0 | 3.5±0.02 | 15.34±0.04 | 36.85±0.13 | 5.96±0.03 | 2.96±0.09 | 59.98±0.23 | 6.95±0.01 | 51.38±0.11 | 31.70±0.07 | 2.54±0.03 | 52.58±0.02 | 13.5±0.11 |
| S10 | 4.53±0.02 | 5.71±0.03 | 2.64±0.12 | 7.74±0.01 | 10.81±0.30 | 17.85±0.01 | 2.13±0.02 | 3.59±0.21 | 32.96±0.01 | 46.05±4.12 | 26.36±0.22 | 48.70±0.11 | 17.38±0.21 | 27.65±0.03 | 18.11±0.21 |
| S11 | 6.1±0.51 | 0.93±0.01 | 0.81±0.01 | 13.88±0.03 | 14.08±0.07 | 17.22±0.02 | 3.19±0.01 | 1.83±0.01 | 37.42±0.09 | / | 73.08±0.09 | 32.56±0.04 | 43.94±0.29 | 27.63±0.14 | 13.31±0.09 |
| S12 | 1.89±0.01 | 2.6±0.07 | 0.92±0.11 | / | 1.49±0.03 | 51.27±0.03 | 4.32±0.08 | 1.61±0.06 | 50.89±0.11 | 3.27±0.03 | 36.67±0.09 | 40.56±0.04 | 2.81±0.03 | 47.59±0.12 | 12.84±0.54 |
| S13 | 6.06±0.07 | 5.99±0.05 | 2.96±0.04 | 6.78±0.01 | 7.72±0.01 | 25.80±0.17 | 2.82±0.02 | 5.79±0.09 | 31.73±0.47 | 26.56±0.83 | 36.98±0.01 | 44.77±0.13 | 15.54±0.05 | 40.94±0.17 | 18.69±0.03 |
| S14 | 6.43±0.01 | 5.11±0.01 | 3.32±0.02 | 5.56±0.01 | 12.23±0.21 | 22.17±1.25 | 2.72±0.02 | 3.39±0.01 | 30.02±0.05 | 16.63±2.14 | 29.89±0.06 | 50.83±0.25 | 19.22±0.03 | 35.6±0.04 | 15.09±0.02 |
| S15 | 5.53±0.04 | 7.12±0.06 | 2.49±0.04 | 8.73±0.18 | 15.46±0.04 | 18.2±0.81 | 1.85±0.04 | 2.83±0.07 | 28.89±0.14 | 50.53±0.26 | 23.67±0.11 | 47.21±0.11 | 20.49±0.12 | 27.10±0.09 | 18.05±0.07 |
| S16 | 12.37±0.10 | 1.69±0.07 | 2.32±0.01 | 8.65±0.10 | 22.33±0.11 | 23.58±1.20 | 3.18±0.03 | 1.9±0.02 | 20.14±0.03 | / | 61.77±0.14 | 20.92±0.08 | 16.22±0.05 | 26.79±0.02 | 10.55±0.02 |
| S17 | 3.53±0.01 | 6.72±0.05 | 1.83±0.01 | 5.61±0.02 | 8.58±0.01 | 37.20±3.45 | 3.42±0.05 | 1.75±0.03 | 40.52±0.03 | 16.28±2.31 | 21.28±0.05 | 37.26±0.25 | 9.34±0.11 | 36.07±0.09 | 12.48±0.03 |
| S18 | 5.56±0.01 | 21.08±0.39 | 3.13±0.02 | 6.46±0.01 | 8.08±0.02 | 18.47±0.12 | 3.53±0.02 | 2.60±0.12 | 36.71±0.16 | 18.04±1.21 | 39.04±0.32 | 29.67±0.07 | 13.41±0.05 | 35.42±0.05 | 46.67±0.01 |
| S19 | 2.28±0.23 | 10.16±0.07 | 1.28±0.01 | 0.66±0.01 | / | 28.07±0.01 | 3.99±0.01 | / | 104.9±0.34 | 13.36±0.11 | 43.13±0.14 | 27.80±0.02 | / | 45.29±0.03 | 40.27±0.11 |
| S20 | 2.39±0.01 | 13.46±0.05 | 1.63±0.01 | 0.71±0.12 | 0.68±0.01 | 11.64±0.06 | 2.25±0.02 | / | 57.26±0.21 | 9.97±0.18 | 24.5±0.11 | 15.16±0.03 | / | 23.95±0.03 | 34.65±0.02 |
| S21 | 2.34±0.36 | 34.12±0.13 | 4.09±0.05 | 3.18±0.04 | 3.06±0.08 | 9.56±0.19 | 1.38±0.12 | 1.29±0.01 | 43.11±2.03 | 15.71±0.06 | 10.58±0.72 | 9.80±0.24 | 3.82±0.05 | 28.75±0.02 | 98.61±0.28 |
| S22 | 5.62±0.03 | 3.06±0.01 | 0.83±0 | 7.93±0.05 | 16.08±0.19 | 38.92±0.16 | 4.09±0.01 | 3.25±0.22 | 44.78±2.34 | 22.84±0.52 | 60.19±0.02 | 38.46±0.11 | 21.48±0.09 | 43.81±0.13 | 21.48±0.02 |
| S23 | 2.86±0.06 | 44.55±0.02 | 5.45±0.31 | 2.27±0.03 | 1.83±0.11 | 2.27±0.01 | 0.39±0.01 | 0.21±0.02 | 34.05±0.13 | 15.48±0.93 | 3.26±0.01 | 2.8±0.65 | 6.39±0.01 | 23.33±0.11 | 111.72±0.29 |
| S24 | 4.44±0.04 | 1.72±0.03 | 0.83±0.08 | 6.98±0.01 | 6.01±0.06 | 24.88±0.58 | 4.52±0.02 | 2.25±0.01 | 73.26±0.22 | 6.43±0.24 | 73.71±0.01 | 37.87±0.02 | 13.36±0.07 | 36.24±0.24 | 17.62±0.08 |
| S25 | 3.11±0.01 | 36.87±0.02 | 4.97±0.05 | 1.34±0.01 | 1.03±0.73 | 7.2±0.02 | 1.4±0.01 | / | 47.06±0.11 | 16.42±0.06 | 13.83±0 | 7.41±0.03 | 5.84±0.12 | 30.4±0.11 | 101.86±2.42 |
| S26 | 2.67±0.02 | 20.91±0.03 | 2.00±0.01 | 0.75±0.01 | / | 20.29±0.09 | 3.26±0.00 | / | 114.32±0.52 | 17.95±0.03 | 37.97±0.04 | 24.88±0.02 | / | 44.62±1.06 | 62.59±0.35 |
| S27 | 1.25±0.05 | 26.03±0.02 | 5.39±0.02 | 2.04±0.02 | 2.56±0.03 | 2.99±0.05 | / | / | 42.33±0.39 | 19.13±0.09 | 7.52±0.02 | 5.26±0.01 | 8.51±0.01 | 30.6±0.16 | 121.39±0.27 |
| S28 | 11.57±0.03 | 2.07±0.01 | 2.18±0.03 | 12.99±0.09 | 13.17±0.29 | 17.81±0.03 | 3.92±0.01 | 2.65±0.08 | 43.03±0.29 | / | 50.21±0.01 | 39.12±0.08 | 24.77±0.02 | 26.91±0.07 | 13.81±0.02 |
| S29 | 4.08±0.23 | 38.66±0.01 | 3.74±0.12 | 5.29±0.00 | 2.94±0.04 | 6.82±0.03 | 1.62±0.01 | / | 50.48±0.06 | 17.29±0.05 | 11.34±0.01 | 9.98±0.02 | 5.55±0.01 | 32.83±0.01 | 114.69±0.41 |
| S30 | 5.56±0.02 | 6.61±0.03 | 2.62±0.07 | 8.35±0.01 | 12.12±0.05 | 20.03±0.62 | 2.14±0.02 | 1.79±0.13 | 33.76±0.31 | 28.01±0.58 | 23.64±0.37 | 35.85±0.09 | 11.19±0.47 | 27.33±0.87 | 11.95±0.04 |
| S31 | 3.36±0.51 | 1.39±0.39 | 0.61±0.11 | 3.97±0.06 | 5.19±0.58 | 14.91±0.01 | 2.24±0.00 | / | 23.36±0.71 | 8.99±0.12 | 39.01±0.26 | 16.31±0.07 | 12.19±0.06 | 23.10±0.11 | 10.43±0.44 |
| S32 | 2.73±0.00 | 4.31±0.03 | 1.89±0.05 | 1.17±0.02 | 4.33±0.12 | 40.18±1.50 | 6.29±0.01 | 2.11±0.07 | 52.58±0.58 | 5.45±0.03 | 37.22±0.07 | 40.89±0.05 | 9.03±0.01 | 45.29±0.52 | 11.85±0.11 |
| S33 | 6.97±0.01 | 4.03±0.01 | 3.13±0.01 | 3.34±0.03 | 8.79±0.06 | 37.55±0.47 | 2.28±0.04 | 3.21±0.05 | 32.79±0.04 | 5.80±0.02 | 29.1±0.11 | 58.7±0.23 | 21.07±0.12 | 38.01±0.05 | 19.51±0.19 |
| S34 | 4.82±0.61 | 1.76±0.06 | 3.13±0.03 | 2.78±0.04 | 7.82±0.05 | 22.46±0.82 | 2.14±0.05 | 2.14±0.01 | 33.68±0.09 | 14.22±0.03 | 28.13±0.17 | 47.08±0.81 | 15.79±0.01 | 22.71±0.03 | 9.14±0.55 |
| S35 | 3.65±0.04 | 7.96±0.01 | 1.85±0.05 | 3.79±0.02 | 5.33±0.34 | 42.41±0.84 | 3.7±0.02 | 2.01±0.06 | 44.08±0.15 | 18.03±0.02 | 20.43±0.06 | 48.56±0.21 | 11.82±0.05 | 42.71±0.07 | 14.7±0.21 |
| S36 | 1.98±0.02 | 0.78±0.02 | 1.12±0.06 | 0.74±0.00 | 2.65±0.03 | 29.14±0.03 | 4.13±0.04 | 3.19±0.07 | 32.02±0.21 | 3.89±0.03 | 35.32±0.07 | 44.79±0.11 | 5.45±0.04 | 42.52±0.03 | 10±0.01 |
| S37 | 2.82±0.14 | 2.59±0.00 | 1.36±0.11 | 1.48±0.04 | 4.25±0.06 | 37.64±0.03 | 6.03±0.11 | 2.80±0.01 | 58.78±0.03 | 5.9±0.01 | 35.24±0.57 | 39.55±0.18 | 7.78±0.09 | 52.17±0.21 | 12.2±0.04 |
| S38 | 0.82±0.13 | 1.59±0.02 | / | / | 0.64±0.03 | 44.89±0.12 | 3.15±0.02 | 1.55±0.07 | 22.35±0.02 | / | 29.14±0.02 | 37.89±0.36 | / | 39.54±0.53 | 14.5±0.05 |
| S39 | 5.26±0.01 | 4.53±0.14 | 1.04±0.01 | 7.35±0.05 | 11.32±0.37 | 18.81±0.51 | 3.83±0.00 | 1.71±0.11 | 31.76±0.51 | 34.26±0.01 | 57.26±0.01 | 36.55±0.08 | 18.7±0.01 | 35.16±0.02 | 20.07±0.09 |
| S40 | 4.93±0.02 | 5.09±0.01 | 2.49±0.01 | 7.18±0.05 | 11.09±0.01 | 16.6±0.18 | 1.75±0.01 | 1.71±0.02 | 30.16±0.08 | 55.58±0.19 | 16.05±0.03 | 40.96±0.02 | 14.45±0.07 | 27.97±0.03 | 17.55±0.06 |
| S41 | 0.67±0.03 | 1.5±0.17 | / | / | 0.64±0.01 | 55.43±0.03 | 3.09±0.01 | 1.76±0.03 | 24.59±0.25 | / | 23.79±0.05 | 38.4±0.09 | / | 41.88±0.62 | 16.11±0.07 |
| S42 | 3.1±0.17 | 20.15±0.02 | 1.98±0.11 | 1.74±0.03 | 2.48±0.03 | 8.5±0.05 | / | 1.44±0.01 | 23.6±0.07 | 7.09±0.01 | 12.48±0.02 | 12.45±0.05 | 10.03±0.07 | 10.59±0.09 | 35.64±0.23 |
| S43 | 6.15±0.01 | 23.39±0.02 | 3.36±0.01 | 5.46±0.21 | 8.24±0.09 | 16.3±0.14 | 2.22±0.02 | 3.21±0.02 | 36.56±0.13 | 16.86±0.01 | 28.67±0.31 | 28.71±0.03 | 9.07±0.23 | 32.50±0.12 | 48.32±1.21 |
| S44 | 5.51±0.01 | 2.42±0.03 | / | 6.14±0.11 | 16.71±0.83 | 36.9±0.85 | 4.06±0.01 | 7.40±0.01 | 33.15±0.16 | / | 66.86±0.54 | 38.39±0.43 | 11.51±0.23 | 41.03±0.03 | 20.38±0.08 |
| S45 | 6.36±0.28 | 6.27±0.01 | 1.32±0.02 | / | / | 32.09±0.03 | 2.49±0.06 | 2.06±0.01 | 43.42±2.51 | / | 82.8±0.33 | 27.95±0.23 | / | 39.68±0.04 | 15.9±0.09 |
| S46 | 4.88±0.11 | 6.96±0.02 | 2.09±0.03 | 11.22±0.10 | 16.92±0.01 | 11.96±0011 | 1.46±0.01 | 2.05±0.06 | 28.31±0.11 | 61.02±0.01 | 19.46±0.12 | 52.91±0.12 | 19.09±0.17 | 21.01±0.03 | 19.5±1.04 |
| S47 | 7.52±0.22 | 4.55±0.03 | 1.12±0.05 | 9.08±0.03 | 15.47±0.25 | 10.55±0.03 | 3.27±0.00 | 3.74±0.04 | 39.27±0.05 | 22.43±0.05 | 51.12±0.32 | 40.72±0.09 | 15.02±0.34 | 29.61±0.01 | 14.75±0.51 |
| S48 | 10.86±0.01 | 1.09±0.97 | 1.60±0.05 | 20.30±0.05 | 19.25±0.17 | 10.3±0.11 | 2.28±0.04 | 1.73±0.01 | 39.57±0.02 | / | 53.04±0.12 | 32.45±0.34 | 49.58±0.12 | 21.09±1.20 | 11.05±0.06 |
| S49 | 5.61±0.06 | 5.22±0.01 | 1.18±0.12 | 8.54±0.08 | 11.50±0.03 | 16.51±0.06 | 3.03±0.01 | 3.58±0.08 | 28.89±0.18 | 34.05±0.02 | 48.05±0.05 | 35.54±0.12 | 17.28±0.45 | 32.02±0.91 | 18.82±0.14 |
